# Cost-Effectiveness of Quantiferon®-TB Gold-In-Tube Versus Tuberculin Skin Testing for Contact Screening and Treatment of Latent Tuberculosis Infection in Brazil

**DOI:** 10.1371/journal.pone.0059546

**Published:** 2013-04-04

**Authors:** Ricardo Ewbank Steffen, Rosângela Caetano, Márcia Pinto, Diogo Chaves, Rossini Ferrari, Mayara Bastos, Sandra Teixeira de Abreu, Dick Menzies, Anete Trajman

**Affiliations:** 1 Internal Medicine Post-Graduation Program, Federal University of Rio de Janeiro, Rio de Janeiro, Brazil; 2 Social Medicine Institute, State University of Rio de Janeiro, Rio de Janeiro, Brazil; 3 Fernandes Figueira Institute, Fiocruz, Rio de Janeiro, Brazil; 4 Health Education M.Sc, Gama Filho University, Rio de Janeiro, Brazil; 5 Santa Casa de Misericórdia do Rio de Janeiro Hospital, Paschoal Granato Laboratory, Rio de Janeiro, Brazil; 6 Montreal Chest Institute, McGill University, Montreal, Quebec, Canada; Johns Hopkins Bloomberg School of Public Health, United States of America

## Abstract

**Background:**

Latent tuberculosis infection (LTBI) is a reservoir for new TB cases. Isoniazid preventive therapy (IPT) reduces the risk of active TB by as much as 90%, but LTBI screening has limitations. Unlike tuberculin skin testing (TST), interferon-gamma release assays are not affected by BCG vaccination, and have been reported to be cost-effective in low-burden countries. The goal of this study was to perform a cost-effectiveness analysis from the health system perspective, comparing three strategies for LTBI diagnosis in TB contacts: tuberculin skin testing (TST), QuantiFERON®-TB Gold-in-Tube (QFT-GIT) and TST confirmed by QFT-GIT if positive (TST/QFT-GIT) in Brazil, a middle-income, high-burden country with universal BCG coverage.

**Methodology/Principal Findings:**

Costs for LTBI diagnosis and treatment of a hypothetical cohort of 1,000 adult immunocompetent close contacts were considered. The effectiveness measure employed was the number of averted TB cases in two years. Health system costs were US$ 105,096 for TST, US$ 121,054 for QFT-GIT and US$ 101,948 for TST/QFT-GIT; these strategies averted 6.56, 6.63 and 4.59 TB cases, respectively. The most cost-effective strategy was TST (US$ 16,021/averted case). The incremental cost-effectiveness ratio was US$ 227,977/averted TB case for QFT-GIT. TST/QFT-GIT was dominated.

**Conclusions:**

Unlike previous studies, TST was the most cost-effective strategy for averting new TB cases in the short term. QFT-GIT would be more cost-effective if its costs could be reduced to US$ 26.95, considering a TST specificity of 59% and US$ 18 considering a more realistic TST specificity of 80%. Nevertheless, with TST, 207.4 additional people per 1,000 will be prescribed IPT compared with QFT.

## Introduction

According to the World Health Organization (WHO), in 2010 an estimated 8.8 million people were infected with tuberculosis (TB), with the disease being responsible for 1.4 million deaths [1]. Even though disease incidence and mortality have been declining steadily, no less than one third of the world population has latent TB infection (LTBI) [2]. To meet WHO's goal of eliminating the disease by 2050, new approaches to reduce this vast reservoir of LTBI are needed [3]. Recently infected individuals have a high risk of developing active TB during the first two years following infection [4]. This risk can be reduced by as much as 90% with isoniazid preventive therapy (IPT) among those who adhere to the full regimen. [5], [6] However, current preventive treatment regimens are lengthy and require close monitoring of side effects, so adherence to a full course of treatment is often suboptimal [5], [7]. Therefore, it is essential to properly identify those individuals who actually have LTBI, and new diagnostic techniques are being evaluated for this purpose.

The most studied and widely used test for the diagnosis of LTBI, the tuberculin skin test (TST), is based on Robert Koch's description of the tuberculin, and has been available for over a century [8], [9]. TST might give false positive results due to previous BCG vaccination and to non-tuberculous mycobacteria (NTM) infection [10], [11], [12]. These are pressing issues in subtropical, high-burden countries, where BCG vaccination is implemented and NTM infections are prevalent [10]. Moreover, a TST might remain positive many decades after infection,[13] and cannot distinguish remote from recent infection, which has a higher risk of progression to active TB. [14] False positive tests will result in more subjects undergoing IPT, increasing costs with follow-up and adverse events, mainly severe drug-induced liver injury (DILI). Finally, TST requires at least two visits, which increase patients' costs and possible loss of result reading.

Newer interferon-gamma release assays (IGRA) have the advantage of using specific *M. tuberculosis* antigens, which appear to provide a higher specificity [15]. However, IGRA tests require equipment and consumables that translate into high costs for the health system.

Studies in high-income, low TB burden countries have suggested that three different commercially available IGRA tests are cost-effective in distinct populations [16], [17], [18], [19], [20], [21], [22], [23], [24], [25], [26], [27]. Many of these countries have incorporated IGRA tests in their routine guidelines [28]. In recently revised guidelines, the Brazilian National TB Program (NTP) recommended screening all contacts in cities where the incidence rate is under 50/100,000 inhabitants, as the national overall TB incidence rate has been reduced to 37/100,000 inhabitants [29]. IGRA tests have not been incorporated in these guidelines because no health economic evaluations of these tests in high-burden countries with broad BCG coverage are available. Moreover, previous cost-effectiveness analyses have attributed a very low specificity to the tuberculin skin test in BCG-vaccinated individuals,[20], [22], [24] although BCG vaccination in infancy, which is the standard practice in Brazil,[29], [30] is unlikely to result in a positive test in adults [12].

The aim of the present study was to perform a cost-effectiveness analysis from the health system perspective, comparing three different strategies for screening and treating LTBI in Brazil: TST alone, QuantiFERON TB® Gold in-Tube (QFT-GIT), and TST followed by QFT-GIT confirmation when the former is positive (QFT-GIT/TST).

## Methods

### Model structure

**Figure 1:**
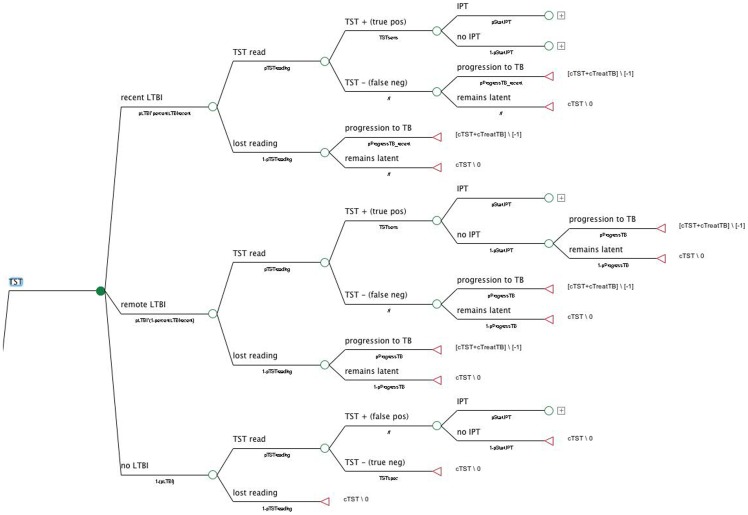
Decision subtree diagram of the Tuberculin Skin Testing screening strategy for LTBI immunocompetent adult contacts.

We conducted an economic analysis considering a hypothetical cohort of 1,000 immunocompetent 35-year-old close contacts of TB index cases followed for two years after LTBI diagnosis, since this is the period when the risk of developing active TB is greatest. A decision-tree analysis using the TreeAge ProTM 2011 (TreeAge Software Inc., Williamstown, MA, USA) was built and three strategies for detecting LTBI in contacts were compared: (i) TST alone, as presently recommended by the Brazilian National TB Program (NTP); (ii) QFT-GIT, the only IGRA presently approved by the National Health Surveillance Agency (ANVISA) in the country; and (iii) TST/QFT-GIT, a strategy found to be more cost-effective in some high-income countries and recommended by guidelines for screening LTBI in many of them [28]. Figure 1 displays the TST strategy subtree of the analytic decision model.

### Model parameters

The proportion of TST-positive contacts, and the sensitivity and specificity of the tests were derived from two systematic reviews [15], [31]. The rate of progression to disease and other parameters necessary for the analysis were based on the existing literature (see Table 1).

**Table 1:** Modeling inputs, assumptions and ranges used in sensitivity analyses for a hypothetical cohort of 1,000 adult immunocompetent contacts in Brazil.

*Input variable*	*Base-case value*	*Range*	*Source*
Prevalence of LTBI	0.35	0.20–0.65	[31], [47]
Proportion of recent infection among contacts with LTBI	0.10	0.10–0.50	Assumption
Effectiveness of IPT	0.5	0.40–0.90	[5]–[7]
Efficacy	0.9		[5], [6]
Adherence	0.55		[7]
Probability of IPT-related DILI	0.012	0.001–0.02	[39], [48], [49]
Probability of hospitalization due to DILI	0.00012	0.00006–0.0002	[4]
Probability of return to TST reading	0.9	0.87–0.9	Assumption
Probability of indeterminate QFT result	0.02	0.01–0.03	[50]
Probability of progression of recent LTBI to TB	0.70	0.40 – 0.80	[14]
Probability of progression of remote LTBI to TB	0.05	0.03 – 0.10	[14]
QFT-GIT sensitivity	0.70	0.63–0.78	[15]
QFT-GIT specificity	0.95	0.94–0.98	[15]
TST sensitivity (> 5 mm)	0.77	0.71–0.82	[15]
TST specificity (> 5 mm)	0.59	0.59–0.80	[15]

Abbreviations: TST, tuberculin skin test; IPT isoniazid preventive therapy; LTBI, latent tuberculosis infection; DILI, drug-induced liver injury; TB, tuberculosis; QFT-GIT, QuantiFERON-TB**®** Gold-in-Tube Test.

### Model assumptions

We assumed that (i) all contacts are asymptomatic and there are no baseline active cases of TB; (ii) there are no HIV-infected individuals in the cohort; (iii) all LTBI and TB cases are from a fully drug-sensitive strain; (iv) 10% of subjects with LTBI and a positive TST were recently infected (TST conversion); (v) 10% of subjects submitted to TST do not return for reading; (vi) all patients diagnosed with LTBI are prescribed IPT after a clinical examination and a chest radiograph to exclude active TB, (vii) patients undergoing IPT who develop DILI are assumed to have completed only four weeks of IPT, which is insufficient to prevent progression to active TB; (viii) IPT is 90% efficient in fully adherent patients and inefficient in the less than fully adherent; (ix) only 50% of patients are fully adherent to IPT; (x) TB cases are managed under directly observed treatment (DOT).

### Effectiveness measure

The number of averted new TB cases was chosen as the measure of effectiveness. We also compared the number necessary to treat (NNT) with each of the three strategies, and those NNTs were incorporated in the treatment costs.

### Costs

The costs for screening and treatment were analyzed from the National Health System perspective, which is responsible for the funding of TB diagnosis and treatment in Brazil. We considered supplies, equipment, and human resources. All costs were converted to USA dollars (US$) at the rate of 1.76 reais/1 US$, the average conversion rate for 2010. No discounting was applied because of the short horizon of the study (two years). Inflation rate adjustment was not performed.

For a rigorous comparison of the strategies, a micro-costing analysis was carried out for both TST and QFT-GIT, since there are no reference values established for QFT-GIT in the Brazilian National Health System. The value of the QFT-GIT test kit was derived from the market price in Brazil.

In addition to the direct costs with tests, screening costs included one medical visit and one chest radiograph (CXR) when TST or QFT-GIT was positive, to exclude active TB. Costs of IPT included 300 mg of daily isoniazid for nine months and five follow-up visits. A partial treatment cost of IPT was considered for those who do not complete treatment and included the initial diagnostic workup, 2 months of isoniazid treatment and two follow-up visits. Costs of DILI were incorporated into the IPT treatment costs, assuming a 1% incidence rate, and included two additional clinic visits, two complete blood counts and liver function tests, in addition to hospitalization costs in 0.01% of DILI cases (Table 2) [4], [32].

**Table 2:** Cost analysis of screening and treatment of LTBI in Brazil, 2010.

Cost components	Base-case value (US$)	Range	Source
**COSTS RELATED TO LTBI DIAGNOSIS**			
Initial medical visit	4.30	2.15–8.6	MOH
Chest radiograph	5.40	2.70–10.8	MOH
**Cost of QFT-GIT:**			
Phlebotomy: Nursing staff time + laboratory technician time	1.60	0.8–3.2	Calculated*
QFT-GIT test kit	42.95	21.5–86	Diagnostics/Cellestis
Consumables (gloves, syringes, needles, box for syringes)	2.34	1.17–4.68	MOH
Laboratory equipment** per patient	1.37	0.69–2.74	Calculated*
**Total cost of QFT-GIT**	**48.26**	**24.13**–**96.52**	
**Cost of TST:**			
Nursing staff time (application and reading)	3.19	1.6–6.4	MOH
Consumables and materials (gloves, syringes, needles, box for syringes, ruler and thermometer with alarm)	2.39	1.17–4.68	MOH
PPD RT23 2UT/01ml	4.90	2.45–9.8	MOH
Laboratory equipment (Fridge for storage of PPD)	0.08	0.04–0.16	Calculated*
**Total cost of TST**	**10.56**	**5.28**–**21.12**	
**COSTS RELATED TO IPT**			
Isoniazid 300mg/day (monthly)	12.03	4.70–18–4	MOH
Liver function test	3.43	1.70–6.8	MOH
Blood count	2.34	1.17–4.68	MOH
Follow-up visits (5)	4.30 (21.50)	10.75–43	MOH
**Cost of DILI:**			
One week hospitalization costs (severe DILI)	315.00	157.5–630	MOH, [32]
Additional consultation (2)	8.60	4.30–17.20	MOH
Additional blood exams (4)	13.70	6.85–27.4	MOH
**Total cost of DILI**	**337.30**	**168.65**–**674.60**	
**Total cost of IPT (including DILI costs)**			
** Partial treatment (2 months)**	**47**	**23.5**–**94**	**MOH, ** [32]
** Full treatment (9 months)**	**141.35**	**46**–**242.7**	**MOH, ** [34]
**Total cost of TB treatment**	**726**	**189**–**1,452**	[33], [34]

Abbreviations: TST, tuberculin skin test; IPT isoniazid preventive therapy; LTBI, latent tuberculosis infection; DILI, drug-induced liver injury; TB, tuberculosis; QFT-GIT, QuantiFERON-TB**®** Gold-in-Tube Test; MOH, Ministry of Health.

* Estimates based on market prices in Rio de Janeiro city.

** includes a ELISA washer and reader, incubator, centrifuge, computer, printers, and laboratory technician time (49 minutes/patient).

Abbreviations: TST, tuberculin skin test; IPT isoniazid preventive therapy; DILI, drug-induced liver injury; QGT-GIT, QuantiFERON-TB® Gold-in-Tube Test, PPD, purified protein derivative 1US$  =  R$ 1.76 (mean exchange rate in 2010).

The costs of supplies and drugs were obtained from public Ministry of Health sources, as were the costs of tests and hospitalization. Costs of follow-up visits for LTBI and TB treatment were derived from previous studies [33], [34]. Equipment costs were valued using the life-cycle cost method [35]. Equipment maintenance was also included in cost calculation. Time logs for each activity were recorded during routine practice. Items and values of costs are detailed in Table 2. The incremental cost-effectiveness ratio (ICER) was calculated using the least expensive strategy as the comparator. [36] Life span was defined as 20 years.

An extrapolation of costs and TB cases averted per year, considering the TB incidence in Brazil and an average of 4 contacts per TB case [31], regardless of the TB incidence rate in the area, was performed.

### Sensitivity analysis

We performed one-way and two-way deterministic sensitivity analyses to test the robustness of results varying the parameters with a significant degree of uncertainty: TST and QFT-GIT sensitivity and specificity, LTBI and TB treatment costs (considering self-administered and DOT), prevalence of LTBI, rate of progression to active TB from recent and remote TB infection, costs of QFT-GIT, IPT completion rate. The ranges are displayed in Tables 1 and 2.

The study was approved by the Brazilian National Ethical Committee (CONEP 572/09).

## Results

Test costs were US$ 10.56 per TST and US$ 48.26 per QFT-GIT, mostly due to supplies (tuberculin and QFT-GIT test kit). Health system costs for screening and treating 1,000 contacts were US$ 105,096 for TST alone, US$ 121,054 for QFT-GIT alone and US$ 101,948 for TST/QFT-GIT (Table 3).

**Table 3:** Effectiveness and total costs (in US$) for screening and treating a hypothetical cohort of 1,000 adult immunocompetent TB contacts in Brazil, 2010.

EFFECTIVENESS	TST	QFT-GIT	TST+ QFT-GIT
Number of TB cases prevented*	6.56	6.63	4.59
Number of people on IPT	482.4	277.5	181.8
Number of LTBI subjects treated to prevent one TB case	73.5	41.9	39.5
Number of extra subjects undergoing treatment	239.9	32.5	12
**COSTS (in US$) per 1,000 patients**			
Diagnostic costs	38,544	74,968	62,029
LTBI treatment costs	45,346	26,085	17,087
Costs with extra IPT	22,551	3,055	1,128
IPT-related DILI costs	529.4	304.6	198.9
TB treatment costs	21,206	20,001	22,832
**Total costs**	**105,096**	**121,054**	**101,948**
Cost per averted TB case	16,021	18,259	22,211

* Considering that no intervention would result in 21 TB cases per 1,000 contacts.

1US$  =  R$ 1.76 (2010 exchange rate).

During the two years following contact, TST alone averted 6.56 new TB cases, QFT-GIT alone averted 6.63 and TST/QFT-GIT averted 4.59. The number necessary to treat LTBI to avert one new TB case was 73.5, 41.9 and 39.5, respectively. The number of subjects undergoing IPT was highest on the TST strategy (482.4), followed by the QFT-GIT strategy, with 277.5 and TST followed by QFT-GIT, with 181.2. The number of subjects undergoing treatment were 482.4, 277.5 and 181.2, respectively (Table 3).

TST was the most cost-effective strategy (US$ 16,021/averted case, Table 3), followed by TST/QFT-GIT (US$ 18,259) and QFT-GIT alone (US$ 22,211). ICER was US$227,977/averted case for the QFT-GIT strategy. The TST/QFT-GIT strategy was dominated.

Considering the Brazilian present incidence of around 70,000 TB [37] cases per year and an average of 4 investigated contacts per case [31], the most cost-effective strategy (TST) would incur in a total cost of US$ 29,406,880 for the Health System and the prevention of 1,837 new TB cases/year in the country. Incorporating the QFT-GIT would cost an extra US$ 4,468,240 to avoid 19.6 extra cases/year.

**Figure 2:**
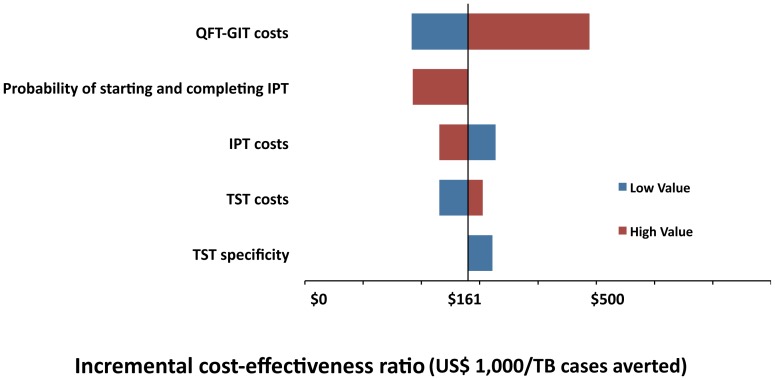
Two-way sensitivity analysis of QFT-GIT costs at different TST specificities. The red-shaded area represents the values where the QFT-GIT only strategy is more costly. The blue-shaded area represents the values where the TST only strategy is more costly. QFT strategy became less costly than TST if its costs were US$ 26.95 considering a TST specificity of 59% and US$ 18 considering a more realistic specificity of 80%.

**Figure 3:**
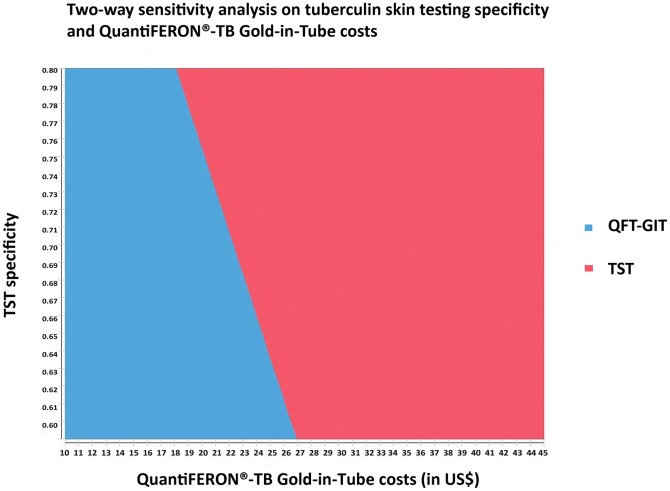
Tornado diagram for Tuberculin Skin Testing versus QuantiFERON® Gold-In-Tube as the screening strategy for LTBI. Termini of bars represent the incremental cost-effectiveness ratio (incremental cost/averted TB case) at the low and high assumption values for the different variables. Longer bars represent parameters to which the model is more sensitive.

In one-way sensitivity analysis, we found two variables to be important: TST specificity and QFT costs. In a two-way sensitivity analysis varying QFT costs and TST specificity, the QFT strategy became more cost-effective than TST if its costs were US$ 26.95 considering a TST specificity of 59% and US$ 18 considering a more realistic specificity of 80% (Figure 2). Other variables did not substantially affect our results and are shown in a tornado diagram (Figure 3).

## Discussion

Newer health technologies often bring new costs, requiring cost-effectiveness studies before they are incorporated into the health system. Their implementation is justified if effectiveness outweighs the higher costs [38]. IGRA tests have replaced or have been added to TST in developed countries, because they appear to be more specific and were considered cost-effective [16], [17], [18], [19], [20], [21], [22], [23], [24], [25], [26], [27], [39]. The present study was designed to evaluate the cost-effectiveness of these strategies in a population in which TST specificity is thought to be lower than the specificity of IGRA. Unlike most of the previous cost-effectiveness analyses, [16], [17], [18], [19], [20], [21], [22], [23], [24], [25], [26], [27], [39] we observed that TST was the most cost-effective strategy. Since the effectiveness of TST and QFT in preventing one TB case is very similar, replacing the TST with QFT becomes a question of relative costs of TST and the additional IPTs versus the costs of QFT. In Brazil, QFT-GIT is bought in the international market at relatively high costs and TST and IPT involve mostly labor costs, which are relatively low.

Our findings are similar to those of only one study, in which targeted testing using only the TST was considered to be more cost-effective than using QFT-GIT or T-SPOT®.TB, although these results were not maintained in sensitivity analyses[40].

The advantage of QFT-GIT in other studies was related to savings with patient evaluation and treatment for TB and IPT. However, health system costs in Brazil are considerably lower than those reported in other countries, and these lower costs had substantial impact in our results. TB treatment costs in our study were derived from the literature [33] and sensitivity analyses considered a range that included costs with self-administered and DOT treatment [34]. Our findings remained consistent in this sensitivity analysis. Regarding IPT costs, TST remained the dominant strategy within the range analyzed (US$ 46-141.35), but QFT-GIT would be more cost-effective if IPT costs were above US$ 332. However, it is unreasonable to suppose that IPT costs would be more than half the cost for active TB treatment. In most similar studies, IPT costs were between 2 and 7% of the TB treatment costs [16], [17], [19], [20], [22], [23], [24], [25], [26], [27], [41], [42] DILI costs arbitrated in our study were low when compared to the literature [16], [17], [19], [20], [22], [23], [24], [25], [26], [27], [41], [42]. Since the probability of severe adverse events in this population is low (young cohort and low adherence), the impact of DILI costs on the overall costs was minimal. Because DILI costs were incorporated into the overall IPT costs, this was accounted for in the sensitivity analysis. Besides costs with DILI, a low adherence to IPT reduces the overall IPT costs.

Another possible explanation for our contrasting findings is the specificity of TST, considered in other studies to be as low as 15% in the base case [22]. There is a general misconception that BCG vaccination has a high impact on TST false positive results. While this is true when subjects are vaccinated after 2 years of age, it is very unlikely that a TST-positive test in an adult contact who was BCG-vaccinated at birth results from that vaccination rather than from LTBI [12]. We used the pooled TST specificity for BCG-vaccinated populations described in a meta-analysis (59%) [15]. However, this meta-analysis included six studies in four countries where BCG is used after infancy [30]. The current practice in Brazil is one vaccination at birth, and the specificity of TST is probably much higher, justifying a range of up to 90% and not less than 59% in our sensitivity analysis. Using a higher TST specificity in this population would make the TST strategy appear more cost-effective than in the base-case scenario.

An universal limitation of economic analyses of LTBI detection and treatment is related to the sensitivity of tests: since there is no golden standard for LTBI, they are based on studies performed in populations with active TB, in whom TST and IGRA tests, which are based on immune response, have probably lower sensitivity [15]. However, this limitation affects equally both tests. Although we considered a higher sensitivity for TST than for QFT, as found in the meta-analysis,[15] the loss of reading of TST decreased its effectiveness.

In addition to the tests' accuracy, previous studies were also sensitive to LTBI prevalence, a parameter with a high degree of uncertainty. Prevalence of LTBI is generally estimated by TST surveys, and there is a very wide variation in the prevalence of LTBI among contacts [31]. We used a large range of LTBI prevalence (20–65%) in our sensitivity analysis, and there was no impact in the final conclusion.

Our study has a few limitations. We did not consider costs for repeated TST in case of lost reading and for diagnosing TST conversion. In addition, transmission of TB (secondary cases) was not accounted for. The option of not including transmission is justified by the short-term horizon and significant uncertainty that this variable can bring to the model. Multidrug resistance (MDR) cases were not considered because the prevalence is still modest in the country (<1%), despite the high rates of treatment default. [29] Finally, we performed a short-term analysis and did not incorporate gain in quantity or quality of life as the outcome measure. The choice of not including utility as an outcome was based on the lack of data on quality of life of TB patients in Brazil.

Generalization for other countries is difficult because local health system structure, financing and salaries as well as other parameters such as TB incidence and HIV-prevalence are widely variable. However, our results show that adoption of new technologies should not be based on apparently robust results on cost-effectiveness from other health systems. They should instead consider each country's scenarios and parameter values.

Conversely, the study has the advantage of considering the range of comprehensive systematic reviews for the most sensitive parameters, named prevalence of LTBI and accuracy of tests.

Health decision makers do not make choices based exclusively on economic analyses. Ethical, equity and protection from harm are important aspects to be considered. Although TST was the most cost-effective strategy, it had a non-monetary cost of treating a high number of subjects. On the other hand, TST is used for more than a century [13] and the benefits from IPT in TST-positive subjects is very well established [43], [44], [45]. Moreover, TST conversion detects recent TB infection, which has the highest risk of progression to active disease in a cohort of close contacts [4]. This is not yet the case for IGRA tests [46]. On the other hand, feasibility is another important issue. Training for TST is complex, time consuming and might incur in extra costs, not considered in the present study.

At present, our analysis suggests that unless a significant reduction in QFT-GIT costs is obtained, TST is currently the most cost-effective screening strategy for LTBI in the Brazilian scenario, despite the high number of subjects undergoing IPT. More studies on the long-term cost-effectiveness of this new technology, considering different subpopulations, are still needed.
